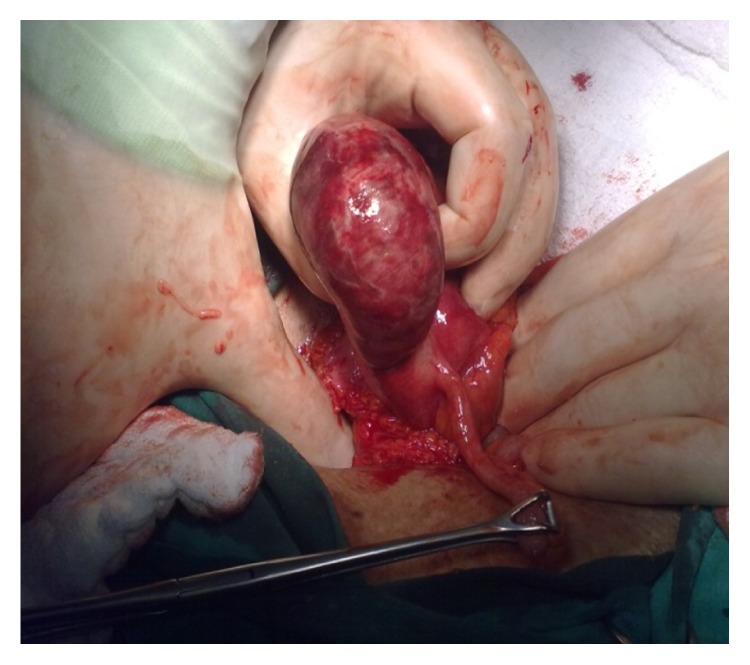# Uncommon Caecum Diverticulitis Mimicking Acute Appendicitis

**DOI:** 10.1155/2016/5427980

**Published:** 2016-02-18

**Authors:** Özkan Yilmaz, Remzi Kiziltan, Vedat Bayrak, Sebahattin Çelik, Iskan Çalli

**Affiliations:** ^1^General Surgery Department, Yüzüncü Yıl University Faculty of Medicine, 65090 Van, Turkey; ^2^Ceyhan Government Hospital, Department of General Surgery, Adana, Turkey; ^3^Van Training and Research Hospital, Department of General Surgery, Van, Turkey

## Abstract

Diverticulum of the cecum is a rarely seen reason of acute abdomen and it is difficult to be distinguished from appendicitis. The diagnosis is generally made during operation. We have presented this case in order to remember that it is a disease which should be kept in mind in cases of right lower quadrant pain.

## 1. Introduction

Diverticulum of the cecum is a clinical entity which was originally defined by Potier in the year 1912 and its frequency is not clearly known [1]. It has a generally asymptomatic progress; however, it may rarely have symptoms such as bleeding, perforation, and diverticular inflammation. Since it does not have a specific clinic picture of its own and due to its localization, it is frequently confused with acute appendicitis. The diagnosis is generally made during operation. We have presented this case in order to remember that the diverticulum of the cecum, which is highly rarely seen and difficult to diagnose preoperatively, is a disease which should be kept in mind in cases of right lower quadrant pain.

## 2. Case

**Figure 1:**
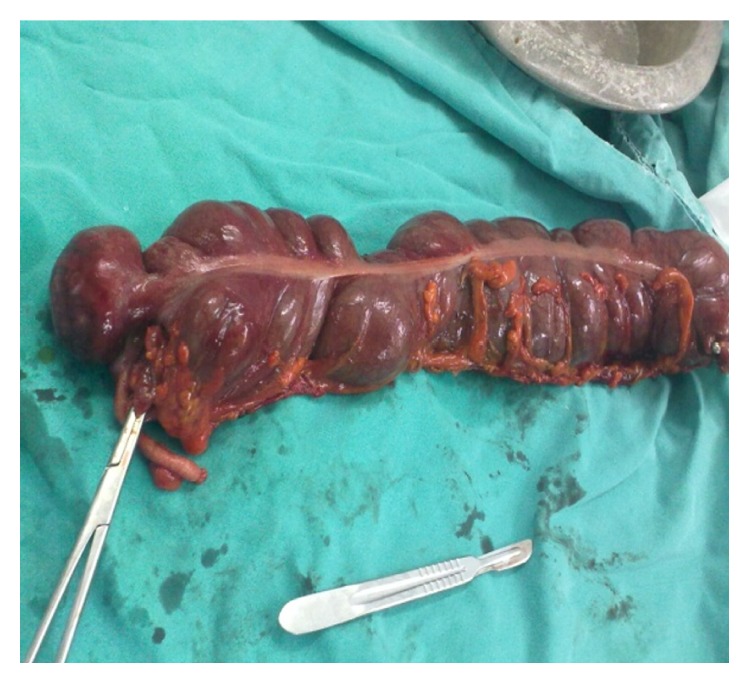


The 73-year-old male patient presented to the emergency service due to abdominal pain. From his medical history, it was learned that his complaints started nearly 10 days ago and were aggravated in the last two days. He had lack of appetite, but no nausea-vomiting. The abdominal examination showed rebound and defense in his right lower quadrant. The biochemical parameters were measured to show CK: 667 U/L CK-MB: 25 U/L LDH: 649 IU/L and the other parameters were normal. The WBC count was measured at 18.600. The emergency abdominal ultrasonography did not show any pathologies in the right lower quadrant other than minimal amount of free fluid. The decision was made to perform emergency operation since the patient had leukocytosis and peritonitis. His appendix was seen to be normal during the operation. The cecum was highly inflamed at a distance of 2 cm to the anterior side and the perforated diverticulum was seen from the top part which had sporadic necrosis. Through the rest of the exploration, no other diverticulitis was observed in the other segments of the colon. Decision was made to perform right hemicolectomy, Figures 1 and 2.

## 3. Discussion

The diverticulum of the cecum is a quite rare condition accompanying appendicitis, which has an incidence of 1/50 to 1/300. It has a higher prevalence in Eastern societies [2]. They are divided into two groups: actual (congenital) and pseudo (acquired). The actual diverticulum develops in the cecum on the 6th week of gestation and it involves all the intestinal layers. Pseudo diverticulum is often similar to sigmoid colon diverticula and the diverticulum does not involve the muscular layer [3].

It has a higher prevalence especially among males and young population (ages 35–45) [4, 5]. Solitary diverticulum of the cecum is located at a distance of nearly 2.5 cm from the ileocecal valve in 80% of the cases and it has been reported to be on the posterior side of the colon in 50% of the cases [6]. In our case, the localization characteristics were also similar.

It can be differentiated from appendicitis with which it is generally confused in clinical terms in that the pain starts in the right lower quadrant, continues at the point where it emerged, and lasts longer and the clinical progress is slower as well as the fact that the concomitant symptoms such as nausea and vomiting are less frequent [4].

In most diverticulitis cases, the clinical symptoms are triggered by obstructions caused by fecaloma as is the case with appendicitis [5]. The diverticular perforations located on the posterior side of the cecum tend to cause peritonitis whereas the posteriorly localized ones cause cecal masses when they are perforated and they may mimic perforated carcinoma [7].

In most cases of cecal diverticulitis, the patients present to emergency services with symptoms of acute abdomen and they are generally assessed as appendicitis by surgeons; thus, they are directly taken to the operating theatre without any further studies taken into account. The application area of barium X-rays, one of the most advanced studies in final diagnosis of diverticulum, is highly limited due to the above-mentioned reasons. Furthermore, the ingress of barium into the abdomen via the perforated diverticulum is an undesired situation [4]. In our case, the ultrasonography image taken was reported to have symptoms indicating appendicitis; therefore, no additional studies were considered as needed and the patient was taken into operation with the prediagnosis of appendicitis.

Today, for the diagnosis of the diverticular disease, the sensitivity and the specificity of the CT scan with intravenous and intrarectal contrast can increase up to 100%. It is very helpful especially for revealing apsis in case there is any [8]. The indications of colonoscopy are suspected cancer, constant or episodic pain in the right lower quadrant, and suspicion of a stenosis or recurrent blood loss [9].

The original Hinchey classification was based on both clinical and surgical findings. With the latest diagnostic tools new approach modalities have developed for diverticulitis. As a result of this many new classifications of diverticulitis have been made. The classification made by Pilichos and Bobotis remains to be one of the most widely used classifications. This classification is as follows: symptomatic uncomplicated disease, recurrent symptomatic disease, and complicated disease [10].

There is no clear consensus on the treatment of the diverticulum of the cecum and various methods are applied ranging from conservative medical treatment to right hemicolectomy [11, 12]. Some authors defended conservative treatment with antibiotics in combination with appendectomy for noncomplicated cases if the diagnosis was made during the operation [13]. However, colectomy should be considered in the presence of complications such as free perforation or localized abscess formation.

Oudenhoven et al. successfully provided medical treatment for 41 cases out of 44 cases of preoperatively diagnosed right colon diverticulitis and only 3 patients received elective surgery during the study they conducted. In 5 patients that received medical treatment, the symptoms were repeated and two of them underwent surgery [14].

Fang et al. recommend aggressive approaches in their study that comprised 85 cases [12]. 40% of these patients received right hemicolectomy and 24 patients received only appendectomy by way of surgery. 29.2% of cases that underwent appendectomy had recurrent attacks of diverticulitis and afterwards 12.5% of the cases had to undergo right hemicolectomy. Urgent right hemicolectomy is preferred if excessive edema and inflammation are present, no malignancies can be differentiated, and multiple diverticula are identified [5, 6, 13].

Furthermore, there are also some authors proposing laparoscopic exploration initially if female patients presenting with right pelvic-lower quadrant pain are scheduled for surgery and if necessary diverticulectomy laparoscopically performed by surgeons experienced in this procedure. Additionally, drainage methods have also been reported [15].

The fact that cases of cecal diverticulum and diverticulitis are rather rarely seen and they generally have an asymptomatic progress should not be a reason for them to be forgotten. Cecal diverticulitis/diverticulum should be considered in cases of right lower quadrant pain that has been present for a long time and continues at the site where it emerged. Even though no consensus has been achieved in treatment methods, the clinical picture of the patient should be the basis of the decision.

## Figures and Tables

**Figure 2:**